# Principlism, medical individualism, and health promotion in resource-poor countries: can autonomy-based bioethics promote social justice and population health?

**DOI:** 10.1186/1747-5341-5-1

**Published:** 2010-01-18

**Authors:** Jacquineau Azétsop, Stuart Rennie

**Affiliations:** 1 Faculté de Médécine Teilhard de Chardin, Complexe Médical le Bon Samaritain, N'djaména, BP 456, Chad; 2 Department of Philosophy, University of Cape Town, Cape Town, Private Bag X3 Rondebosch7701, South Africa; 3 Department of Social Medicine, University of North Carolina School of Medicine Chapel Hill, 333 S Columbia Street MacNider Hall, Room 348, CB 7240, Chapel Hill, NC 27599-7240, USA

## Abstract

Through its adoption of the biomedical model of disease which promotes medical individualism and its reliance on the individual-based anthropology, mainstream bioethics has predominantly focused on respect for autonomy in the clinical setting and respect for person in the research site, emphasizing self-determination and freedom of choice. However, the emphasis on the individual has often led to moral vacuum, exaggeration of human agency, and a thin (liberal?) conception of justice. Applied to resource-poor countries and communities within developed countries, autonomy-based bioethics fails to address the root causes of diseases and public health crises with which individuals or communities are confronted. A sociological explanation of disease causation is needed to broaden principles of biomedical ethics and provides a renewed understanding of disease, freedom, medical practice, patient-physician relationship, risk and benefit of research and treatment, research priorities, and health policy.

## Introduction

Respect for autonomy or respect for persons has tended to be the leading principle of biomedical ethics or research ethics, respectively. This principle historically has its roots in the liberal moral and political tradition of the Enlightenment in Western Europe. Within this tradition, the ethical justification of actions or practices strongly depends on the free decisions of individuals, i.e. an action or practice can only be ethically justified when undertaken without any coercive influence and entered by free and informed agreement. While there have always been disagreements on the details, all theories of autonomy agree on two essential conditions: the first is liberty, specifying the independence from controlling influences; the second is agency, referring to the capacity for intentional action[1]. Used in clinical ethics, autonomy functions primarily to examine decision-making in health care and serves to identify actions that are protected by the rules of informed consent, informed refusal, truth telling, and confidentiality[1]. Autonomy-based approaches are strongly expressed in Tom Beauchamp and James Childress' classic text *Principles of Biomedical Ethics *for clinical bioethics and, for research ethics, the influential *Belmont Report*[1,2].

Many criticisms of autonomy-based bioethics have appeared over the past thirty years from a number of different angles, such as feminism, casuistry, disability rights, multiculturalism, cultural studies, and ethnography. In this article, we take a different approach by exploring what we will call the 'medical individualism' that autonomy-based bioethics largely assumes, and by raising questions about the relevance and impact of autonomy-based bioethics in developing countries (and communities within developed equitable ones), especially in light of initiatives to 'build capacity' in research sites and to ensure access to healthcare in resource-poor settings. This paper argues that the medical individualism underlying autonomy-based bioethics renders the latter incapable of addressing some of the most pressing bioethical issues in resource-poor settings, which have to do with social justice. The first section of this paper considers some of the limitations of principlism. The second section examines the inability of this approach to address social justice concerns in resource-poor countries. Finally, the third section attempts to offer an alternative approach by exploring the contribution of the sociological model of disease causation to research ethics, health justice and health policy.

## A brief anatomy of autonomy-based bioethics

One of the major defenders of the centrality of autonomy in bioethics, the British medical ethicist and pediatrician, Raanan Gillon argues that respect for autonomy should hold a primary place among the four principles of biomedical ethics[3]. Other proponents of autonomy, Beauchamp and Childress, define autonomy as a form of personal liberty of action where the individual determines his or her own course of action in accordance with a plan chosen by himself or herself[1]. In application to clinical medicine, respect for autonomy dictates that patients with decision-making ability have a right to voice their medical treatment preferences, and physicians have the concomitant duty to respect those preferences[4]. Like Beauchamp and Childress, Gillon embraces a Millian understanding of autonomy, understanding it as deliberated self rule; the ability and tendency to think for oneself, to make decisions for oneself about the way one wishes to lead one's life based on that thinking, and then to enact those decisions--is what makes morality--any sort of morality--possible[3]. Given its supreme ethical importance, autonomy is not merely a value to be respected, but a virtue or trait that ought to be actively developed, nurtured and promoted.

According to Gillon, other ethical principles (beneficence, non-maleficence, and justice) presuppose (and can be reduced to) respect for autonomy. Beneficence and non-maleficence toward autonomous moral agents presuppose respect for the autonomy of these agents even when they choose to refuse medical interventions which are life-saving. Gillon also takes an autonomy-centered approach to justice, arguing that responding to people's needs justly will require respect for those people's autonomous views, including autonomous rejection of offers to meet their needs; and, more importantly, because providing for people's needs requires resources, including other people's resources[3]. To conclude his praise for autonomy, Gillon writes that respect for autonomy contingently builds in a *prima facie *moral requirement to respect both individual and cultural moral variability[3]. While it is true that not all autonomy-based approaches in bioethics take the explicit and extreme form expressed by Gillon, autonomy continues to be treated implicitly as a primary value in many controversial clinical and research debates, from end of life issues (such as the Terri Shiavo case) to questions of exploitation of research subjects in international health research. When ethical principles conflict, it is often thought that the conflict can be resolved in an ideally impartial way by asking, for example, what the patient wants (or would have wanted) or whether the research subject really understood and freely consented to the procedures described in the research protocol. In this way, the multifarious values involved in the practice of medicine and biomedical research tend to be reduced to the principle of respect for persons, itself narrowly understood as respect for autonomy. Furthermore, the preeminence of autonomy as an ethical value within bioethics is deeply related to the increasing commoditization of medicine in developed countries. For the more that medical practices are justified by reference to patient choice, the more that patients will be viewed as 'clients' and health care professionals perceived as 'service providers'. This model of patient as 'client', which is prevalent in the United States of America and some parts of the western world, assumes affluence and power: the (literate) patient has to be capable of understanding and rationally weighing his/her options--possibly even in disagreement with the physician--and be in a position to pay in exchange for services chosen.

### Autonomy, exaggeration of human agency, and ethical pluralism

An autonomy-based ethics places the responsibility for medical decision-making largely in the hands of the patient. This raises the descriptive question of whether this conception accurately depicts how clinical decisions are actually made, as well as the normative question about whether such a conception of responsibility should (or should not) function as a universal ideal. In regard to the descriptive issue, patients in resource-poor settings are often not concerned with their ability to determine and shape the course of cure. Their arrival at the local health center is the outcome of a long family discussion that led to the collection of money. Sometimes, the patient arrives at the dispensary when the disease has reached its critical stage because the cost of care is too high. The primary expectation of both patient and family is to get the medicine or undergo a medical procedure they need and go back to their workplace. Spending time at the hospital means loss of earnings for them and their families or the diminishment of financial resources. When people can barely afford the cost of care or satisfy the nutritional requirements for a good recovery, the ethics of medical encounter should be understood differently and expressed in different terms than patient choice. Instead of developing a highly-organized medical bureaucracy that cares for the enforcement of patients' rights and protects medical professionals from accusations of malpractice, it would be more helpful to develop new sets of values that guide medical practice and promote patient participation in the healing relationship. The framing of these values may encourage and foster a non-confrontational relationship between health professionals and patients in the clinical setting, and include social challenges that influence health in the bioethics agenda. The role of bioethics will then consist in identifying social values and laws that may guide clinical work, restore the social dimension of medicine, connect the macro-determinants of health to medical practice and health system delivery, avoid the fragmentation of healthcare, and advocate for good health policies.

The challenge facing bioethics in resource-poor settings is not then to mislead people with unrealistic promises of autonomy that very few people can indeed achieve, but to articulate moral principles and societal values that are oriented around the promotion of equitable access to care and which broaden the goals of medicine and public health. The goals of medicine cannot be confined to the alleviation of suffering within the clinical setting. Medicine needs to be concerned with the determinants of good and bad health outside the clinical context in order to contribute to evidence-based clinical and public health interventions and education. The major bioethical questions prevalent in resource-poor countries do not essentially revolve around the provision of informed consent at the individual level, but rather around the burning social questions of access to care, commodification and quality of medical care, the relationship between income disparities and health inequities, the impact of poverty and underdevelopment on population health, priorities in biomedical research, and impacts of gender discrimination on women's health[5,6]. Once the focus is shifted away from the individualistic 'patient as client' paradigm, the social problems connected with the domination of medicine by market forces become apparent. If the goal of medicine is to restore health functioning, bioethics should avoid adopting a conception of autonomy that can be used to justify the domination of healthcare delivery by market forces alone and (wittingly or unwittingly) legitimizing health care systems that exclude the needy sick because the latter are unable to pay (or co-pay) for services or afford hefty medical insurance premiums. Even those bioethicists who promote market-driven medicine based on a libertarian anthropology[7,8] ought to carefully articulate alternative ethical values for health care and biomedical research, if they not to be lured into a 'self-defeating' conception of medicine. As an example of the latter tendency, Robert Sade considers medicine as a market commodity and understands medical practice as sets of skills that physicians are entitled to sell on the marketplace to make as much money as possible. Even the cries of the destitute sick or government regulatory function cannot restrict the physicians' appetite for greater financial reward. Sade's anthropology and approach to medicine is based on the assumption that individuals have the right to select the values that they deem necessary to sustain one's own life. They are also entitled to exercise their judgment to take the best course of action to achieve chosen values. Finally, they have the right to dispose of those values, once gained, in any way one chooses, without coercion by other men[7]. Similarly, Tristram Engelhardt protects human freedom to the point of ignoring the fact that the concern that we have for each other makes life in society possible. For him, as long as freedom functions as a side constraint, and as long as the moral community is based on respect for freedom and not force, individual persons will have the possibility of holding entitlements[8], Engelhardt's suggestion is paradoxical because, in trying to protect freedom of individuals to use their resources to access health care and other goods, he does not ensure that those with few resources have the freedom to obtain health care. Realistically, a genuine affirmation of autonomy cannot result in action informed or motivated by the desire to avoid being a responsible member of one's moral community[9]. Here, responsibility means that one should not exploit others by using autonomy as a warrant to market-driven medicine or profit-seeking attitudes. Once medicine is understood as a commoditized product like any other, those who cannot afford services are merely unfortunate consumers. In this way, a strong emphasis on autonomy can contribute to a culture in which healing and health promotion are no longer at the center of clinical practice and biomedical research.

One can hardly refute the fact that complex social and economic forces have placed patient autonomy at the center of medical ethics, and thereby undermined the age-old ethic of physician beneficence[10]. This change is sustained by waning trust in the traditional patient-physician relationship. With the control of medicine by the forces of the market, patients have become consumers of a market commodity called medical care. As a result of this change, the clinical relationship between the patient and physician begins to be seen as a contract and not as a covenant of care as it was in the past. Autonomy-based bioethics has a tendency to distort the relationship between individuals and the world. On the one hand, it exaggerates the power and range of individual agency; furthermore, it underestimates the impact of society, culture and environment, both on individual decision-making and on health. If persons are regarded as atomistic, certain defensive notions of individualistic rights-based autonomy prevail. If a relational construction of personal identity is employed instead, then respect for autonomy becomes part of a wider morality of relationship and care[1]. 'Atomistic autonomy' is divisive and lacks social rootedness while relational autonomy brings about trust and communality. The second version of autonomy, which reveals our true self in society, presents the possibility of placing trust and partnership at the center of the patient-physician relationship. With such an understanding of personhood, bioethics can better balance its concerns over choices and actions with those of relationship and responsibility. A more plausible philosophical anthropology would conceive individuals as entangled in the world, both capable of acting on it and subject to being affected by it.

Reflection on the notion of disease, both infectious and chronic, can contribute to a more plausible philosophical anthropology for bioethics. Infectious diseases question our understanding of autonomous agency in two important ways. First, as both a victim and a vector, a patient cannot be simply seen as a rational agent who has the final ethical word on his own decisions. Both vulnerability to infection and threat of transmission to others should shape our understanding of patient agency. Second, the concept of choice that shapes our conception of agency in bioethics can no longer be understood in isolation from society. Risk of acquiring and transmitting infectious diseases reflects the patient's interconnectedness with others and the biological environment, an interconnectedness which is always there even when infectious disease is not present[11]. Although the values and desires of the patient obviously need to be considered, the ideal of the autonomous agent will remain a fiction unless the social context of the patient's vulnerability is also considered. For other reasons, chronic disease also challenges our understanding of autonomy, especially when the patient finds it hard to manage his or her chronic condition. Family or friends stand as important resources for decision-making and long-term daily care for chronic diseases. We should then recognize that the family and community, which may play an important role in patient care, are part of the resource needed by the patient to exercise agency[12]. More and more, it is becoming obvious that the promotion of patients' agency requires serious consideration of patients' best interests in a broader way. Against the backdrop of contemporary institutional medicine, family solidarity is more important than ever to help maintain patient's dignity and agency throughout stressful time[13]. Exclusion of family and relatives from the sphere of decision-making on account of respect for individual autonomy does not necessarily serve patients' best interest. Furthermore, primary care, because of its focus on treatment and prevention of chronic and infectious diseases, is the domain of medicine that goes beyond techno-medical solutions to consider patients as persons with their stories, relationships, and social environment in which they live. Consequently, primary care should essentially rely on socially-grounded values rather than on desocialized principles[14].

Family and social relationships are important in the context of clinical medicine. However, we cannot undermine the importance of individual freedom. We simply reject strong claims that do not have any social rootedness. It would be almost unsound and socially untrue to radically endorse autonomy to the detriment of an ethic of responsibility and socially-based care because they are mutually interdependent, and a complete account of medicine's moral axis requires that they be integrated. This reorientation is crucial for reasserting the ethos of clinical medicine, whose fundamental mandate remains the care of others[10].

### Autonomy ethics and the 'moral vacuum'

For Immanuel Kant, respect for persons never refers to the freedom to be left alone. Kant's understanding of respect for autonomy provides the ground for the categorical imperative, which he formulated in five different ways. The third formulation, ".act so that you treat humanity whether in your own person or in that of another, always as an end and never as means only" [15] cannot be reduced to the respect for autonomy often found in the bioethics literature. The view of autonomy commonly found among individuals and in some of the bioethics literature in North America or Western culture is more in tune with John Stuart Mill's formulation of liberty: do not intrude on the freedom of any person by an invasion foreign to his or her own wishes and values. When Kant talks about autonomy, he does not imply that one should act according to one's own desires, unconstrained by a balanced consideration of one's situation as a being-among-others[9] Instead, he refers to the dignity of humans who are capable of making for themselves and others universal law. Hence, autonomy, rightly construed... results in action informed and motivated by the desire to be responsible member of one's moral community (the ground of one's being-among-others)[9]. Kantian autonomy is tied the moral agent's search for the truth and respectable conduct. The autonomous subject does not act in accordance to his or her primary inclination. Kantian autonomy is applied to actions performed when the will is freed from any selfish determination. When humans treat each other as ends and never as means merely, there arises a systematic union of rational beings under common objective laws. Physician and patient, each with their own needs, desires, capabilities, must find those principles that allow them to coalesce into a helping alliance to achieve a common goal.

Contemporary readings often accept a Millian version of autonomy that is associated with self-seeking attitudes. This approach to respect for autonomy refers to the capacity to act on needs, wants, or wishes; a capacity shared by many creatures. Since the person's action is informed by instrumental reasoning, it constricts the scope of reason so that it is subject to any desire or disposition that one happens to endorse at the time one acts[9]. Focusing essentially on individual choices sets up a false and pernicious opposition between persons and the community to which they belong. It is reasonable, on both conceptual and empirical grounds, to suppose that individuals acquire their values through engagement with a concrete moral tradition, rather than through a private and self-directed process. Instead of providing ethical decision-making with an objective and rational process, the obsession with individual autonomy tends to create what McCormick calls a 'moral vacuum', i.e. the disappearance of the network of shared and established goods and values that make the choices of individuals right or wrong, moral or immoral[16].

### Balancing autonomy and community in ethical decision-making

It is hard to undermine the influence of social, cultural and environmental factors on moral decision making. We have to take these factors into account in order to fully appreciate the moral dilemmas and health challenges in settings and traditions where individualism does not prevail. Writing from their Jewish background, Barth-Rogers and Jotkowitz note that within Jewish tradition, the idea of unlimited human autonomy is not a defining value; Judaism deems the intrinsic human value of each individual's life to take precedence over patient autonomy[12]. Similarly, the Confucian culture from East Asia understands the person not only as a rational, autonomous being but also as a relational and altruistic entity whose self-actualization involves participating in and promoting the welfare of fellow persons[4]. In the same line of thought, African traditions present a view of the human person that is essentially relational; it is within the social network that the individual lives and acts as a free person. The Jewish, Confucian, and African cultures convey an understanding of the human person and society which is different from individualism operative in some cultures.

This is where the shortcomings of Gillon's autonomy-centered conception of bioethics become the most obvious. Gillon does not reject the view that particular cultures should be respected, instead he theorizes that the *prima facie *nature of autonomy requires that both the individual and cultural moral variability be respected[3]. But this sense of respect for culture does not adequately reflect the social rootedness of the human person. Despite making 'concessions' to culture, Gillon continues to view societal relationships, determinants and influences to be peripheral to human reason and, because of the danger of ethical relativism, something to be transcended by a universal ethic. Hence, the four principles (with autonomy as supreme among them) can account for all our moral worries and being applied straightforwardly to all situations and contexts[17]. Gillon contends that any other moral principle or value can be explained by one or some combination of the four principles. In fact, however, Gillon's quest for a universal discourse is nothing more than the promotion of one approach to ethics among others, one which reflects specific cultural assumptions concerning individual choice and future-oriented action that are associated with class position and social opportunities and foreign to the lived reality of the poor, the marginalized, and people of color in a multicultural society like the United States[18]. Any attempt to universalize an ethnic particularity fails the test of respect for pluralism in bioethics and in our ever-globalizing world.

In resource-poor countries where medical paternalism prevails on account of patient beneficence and shared-responsibility for health promotion[19], the necessity to create the conditions that improve, for example, patient-physician communication in ways that favor patient agency needs to be acknowledged. Very often, the physician does not even tell the patient what is going on with his or her health. However, the one-sided view of the human person which prevails in autonomy-based bioethics should not be adopted as a model to correct paternalism; a more fruitful alternative would be a combination between a community- and tradition-oriented view and autonomy that conceives decision-making as guided by important human values such as partnership, trust and solidarity, in addition to autonomy. This view would acknowledge the embedded and relational nature of human choices, behavior, ways of expressing emotions and feelings, patterns of thinking, and conceptions of disease and healing.

## Autonomy, biomedical individualism, and social justice

Some criticisms of autonomy-centered bioethics have been purely conceptual. Others have emerged from reflections on its limitations in dealing with collective macro-problems including social, sanitary and environmental problems that mark everyday life in poor countries. Autonomy-based bioethics fails to engage the lived worlds of diversely constituted and situated social groups, particularly those that are marginalized[18]. Similarly, in clinical medicine, broad issues such as the common good, distributive justice and the spirituality of the patient are ignored for the sake of the primacy of secular business concerns. To guide clinical practice, laws have been developed to reduce risk for malpractice and protect patients. However, emphasis placed on the principle of autonomy has led to an excessive control of clinical practice by judicial institutions. Consequently, this obsession with the law has led to the elimination of a wide range of moral concerns from public consideration[16]. To emphasize this point, McCormick criticizes clinical ethics for being preoccupied with cost control that focuses narrowly on matters of financial efficiency, thus exiling the more basic ethical questions (ends of medicine, the meaning of life, death, illness and health)[16]. Furthermore, any public health intervention that adopts the biomedical model fails to address issues of wider social injustices that are responsible for health-related vulnerability and risk.

### Autonomy ethics and medical individualism

The biomedical model is premised on individualism, because it adopts an abstract view of the body and mind of an individual person from a liberal model of economy and politics[20]. In this model, individuals choose health behaviors. Thus, poor health is largely due to exposures to health risks that the individuals have decided not to avoid. This approach to health risks disregards the role of social structures in structuring the array of risk factors that individuals are supposed to avoid[18], and fails to explain how social inequalities can be embodied in poor-health outcomes[21]. Thus, autonomy-focused bioethics, rather than presenting an objective perspective, deprives itself of theoretical tools to adequately address non-pathological causes of ill-health. Similarly, in research sites, much effort is often invested in securing the informed consent of individual participants while often ignoring the broader issues of justice in places where research takes place[22]. Consequently, the absolutization of autonomy with the unreal and distorted picture of the person helps explain why so much bioethical writing is concerned with procedures that protect choice, rather than more substantive issues, with consent itself rather than what is consented to[16]. This tendency to make the social causes of poor health (and the broader ethical problems related to health improvement) invisible can even be seen among those working in public health to the extent that they subscribe to the biomedical model[20].

### Biomedical model and the social gradient in health

Health differentials between individuals cannot be explained simply by their health behavior or lifestyles, but also by their social position and economic status, the social networks to which they belong, and the levels of education that provide them with the means to avoid health risks, deal with adversity, and have access to life-protecting information. The pervasiveness of the social gradient in health remains even when well-designed public health interventions are implemented. Even when these public health interventions may reduce health risks and mortality, they do not eliminate the social gradient because individuals in the lower socioeconomic groups take less advantage of health interventions than those who are better off.

When we compare the health statistics between poor and rich within countries or between countries, the differentials are striking. HIV/AIDS statistics provide us with striking examples of the impacts of socioeconomic status on risk differentials and chances of survival between groups within countries and between countries. Even in developed countries, the geography of HIV/AIDS challenges us to investigate the social causes of its distribution. Risks and survival differentials prompt us to consider a view that places political-economic critiques of global resource distributions, and criticism based on the higher and qualitatively different disease burdens in poor countries within a common framework of international and internal socio-economic structure[23]. At the local level, income inequality in poor countries affects health and can be an indicator of life expectancy[24,25]. Poverty affects individuals' ability to have access to goods which are instrumental for well-being. At the country level, poverty limits government's ability to found social programs and provide people with basic social goods such as safe drinking water, electricity, good public health coverage, healthcare institutions, schools, social services, and economic opportunities. These structural causes are steady and they include access to basic resources that can be used to avoid all sorts of health risks or reduce the negatives outcomes of diseases when they occur[26].

Most public health interventions focus on individual risk factors and behavior. To lessen vulnerability and risk, health professionals will need to address income differences between individuals and population groups. Otherwise, they will only address the symptoms and not the root-causes of poor health. As public health practitioners and other health professions 'resocialize' their conceptions of health and disease, bioethicists should join and inform their efforts. A sociological approach to disease can increase the social relevance of bioethics because it provides an acute perception of disease etiology and pathology that includes the social and material conditions in which people live.

## Sociological model and autonomy-based bioethics

To underscore the difference between Western and non-Western conception of illness, Bowman writes that most non-Western cultures tend to perceive illness in a much broader and far less tangible manner. Illness is often viewed as being linked to social, spiritual, and environmental determinants[27]. The sociological model of disease explanation shares some important connections with many non-Western cultures in which disease representation and explanation is not primarily understood in biomedical terms, but in social ones. Autonomy-based bioethics is premised on the view that disease is located in the individual. The focus on the individual person often reduces the scope of justice in clinical medicine and health research to an equal treatment of individuals involved and a fair distribution of available resources and burden regardless of people's social status, age, race, gender or religion. In the clinics, for example, justice requires that patients whose circumstances are the same deserve the same level and quality of care.

Conversely, the sociological model perceives the disease as an integrated social-physiological process which includes the person's relation to the environment. In addition to its bio-physiological dimension, a disease is a relational phenomenon; as a subjective and socially-constructed reality, a disease develops out of the omnipresence of symptoms and bodily feelings in everyday life. The sociological model allows us to develop a socially-relevant approach to health justice, a new set of principles that may guide research as well as an approach to health policy based on the features of the site where research is done. Thus, this model points to the fact that there are two reminders of our embeddedness in the world relevant to bioethics: first, biological embeddedness and infectious disease and second, social embeddedness, particularly (but not exclusively) in contexts where people are obviously dependent on one another and traditional behavior and customs are strong.

### Contribution of medical sociology: Sociological model and social justice

The current formulation of ethical principles as they are applied to medical research in poor countries is inappropriate for capturing some crucial implications of medical research since they ignore the roots of health crises with which these countries are confronted[28]. Analyzing the health crises in African countries in the late 1980s, the Cameroonian sociologist Jean-Marc Ela argues that disease and malnutrition never exist by themselves; rather they come from a system characterized by violence, by a pattern of impoverishment of the majority, and by the monopoly by a minority of the means to live with dignity[29]. Health interventions should not merely address the symptoms of a disease-producing society, but also its structures. Social structures not only shape distribution of disease across population, but they also determine societal and individual responses to suffering. When the major determinants of health are far from being addressed by a conceptual framework that prioritizes individual problems and morality, there is a need to call its relevance into question. The high rates of infectious diseases in poor countries are linked to poor living conditions and structural problems. These primary sources of exposure and vulnerability to health hazards should necessarily be considered in any attempt to develop bioethical standards for research or any bioethical agenda. The poverty that permeates all spheres of society should be studied because poverty never exists in isolation from societal influences, but rather is integrally a product of the inner workings of each society's political economy. Minimizing the contribution of poverty to the production of disease and disability in poor countries makes suffering invisible and limits our understanding of the etiology of disease.

Medical sociology scrutinizes patterns of diseases and pathways through which social inequalities are embodied in individual vulnerabilities and major epidemics. Thus, the model of disease causation that comes from sociological investigations challenges us to move beyond the clinics or research sites to broaden the scope of justice. Similarly, the prevalence of infectious diseases in resource-poor countries challenges the way justice is understood in research sites. If we consider the patient as a potential victim and vector, we need to shift our gaze from the healthcare that might be most desirable for the individual patient to broader social concerns and the worldview distribution of care that might enable all to achieve opportunities over a reasonable life span[11]. The extension of care to all not only aims at serving individual needs for care, but more importantly it addresses infectious diseases as a threat to population health. Opting out from an intervention of this kind would simply mean that the individual remains a threat to the entire population[3].

The sociological explanation of disease incorporates a distinctive view of etiology, prevention, pathology, treatment, and justice. This approach to disease explanation tacitly promotes a conception of responsibility for infection or disease causation which is not only individual. This approach questions the uses of individualism as methodology and framework for analyzing disease occurrence, and thus criticizes the one-sidedness of the anthropology that sustains the biomedical model.

### Sociological model and justice in current biomedical research

Documents such as the *Declaration of Helsinki *issued by the World Medical Association and the *International ethical guidelines for biomedical research involving human subjects *(CIOMS) as well as the work of the National Council on Bioethics in 2002 and that of the National Bioethics Advisory Commission (NBAC) in 2001 all take material poverty as the main reason for developing bioethical standards that apply to medical research conducted in poor countries. Surprisingly, the bioethics standards they promote hardly reflect the physical, social, and cultural environment of poor countries. This is another important area for revision[28].

Given the substantial differences in individual exposure to health risks and the availability of health protective resources as well as differences in the disease burden and mortality and morbidity at the population level, it is clear that illness in poor countries can be better understood using a 'social causation of illness' perspective. The principles of respect for persons, beneficence, and justice that shape the *Belmont Report *are all built on the biomedical model. The principle of respect for persons reinforces individual agency and protection in the research setting by ensuring that participants are properly informed about the research or the course of care that will be taken to restore normal functioning. The principle of beneficence extends the latter by insisting that research protocols should maximize potential benefits and minimize harm. Finally, the principle of justice ensures that those with diminished autonomy are protected and that participants share in the benefits of the research. Agency, benefit, participation, risk, and vulnerability are all understood from the standpoint of the individually-focused disease management whether in the clinical setting or the research site. To be of broader global significance, ethical principles of biomedical research should be responsive to the context of poverty and social inequities, since these structural factors can lead to increased vulnerability and exploitation. For example, the incapacity of poor people to satisfy their basic needs can lead to increased participation in clinical trials without true understanding of risk and benefit at least in part due to financial incentives. Thus, even if these people 'consent' to participation in a trial, is that decision truly autonomous? It is then clear that 'research protections' cannot be ensured solely through the use of the consent form and the provision of information to the subject. A formal provision of consent by the research subject can simply mask the misery that inhibits his or her ability to consent freely.

Similarly, what counts as 'benefits' can be tied to different levels of poverty and disease burden in different resource-poor countries. Ethical principles and guidelines that oversee biomedical research can be defined in terms of public good rather than merely as an improvement in individual health status because public good and social policy transcend the framework of individual-based ethics[28]. In resource-poor countries, death-rates are high and infectious diseases contribute significantly to the burden of disease--as opposed to richer countries, where cardiovascular disease and cancer are the leading causes of mortality--the difference in exposure, health risk, mortality, and morbidity between poor and rich countries challenges us to develop a new approach to the concept of benefit in biomedical research. We need to think of 'benefits' as running to the whole community in which research takes place, and not just to single research subjects. Therefore, the availability of and access to modern health services is a substantial issue for evaluating the impact of biomedical research benefits in poor countries since the outcomes of health initiatives are largely determined by some structural arrangements that transcend the benefits of research subjects. These arrangements are based upon national and international patterns of control over society's resources.

Current ethical guidelines continue to be inappropriate because they do not address the international context of exploitation within which research is done. People's health status cannot be separated from the capitalist system of resource distribution and exchanges which favors the rich countries or high socioeconomic groups and reinforces the impoverishment of the poor ones. The economic exploitation that prevails in the capitalist system shapes the global and local distribution of resources and diseases as well as the health risks and vulnerability of those who live on the margins of the global market. The concepts of 'benefit' and 'justice' have been inadequately extended to biomedical research in poor countries because the possibility of exploiting the underprivileged is more complex than an exploitative relationship with vulnerable populations in developed countries, where at least the rule of law and the respect due to every citizen have already been institutionalized. Furthermore, the number of research studies conducted in poor countries is increasing because regulatory measures are often less strict; this situation may facilitate the exploitation of the poor, non-respect for basic ethical standards, and unlimited search for benefit.

Bioethics scholarship that focuses on the sociological model considers local as well as global issues of social inequality, because this model is premised on the intimate connection that exists between social inequality and health inequality. The distribution of illness is likely to reflect the geography of inequality. A social approach to bioethics emphasizes distributive justice and benefits at both the population and individual level. Three important principles flow from this analysis. The first one can be called principle of public benefits (community-based approach to benefits); it is a context-based principle which derives from factors that contribute to ill-health and vulnerability to preventable diseases in poor countries. It states that risks, benefits, and equity can no longer be defined in terms of individual health, but also in relation to the international, national and local contexts[23]. Such a principle challenges the individualistic understanding of benefits in places where exploitation and inequality are at the center of research. Consequently, a community-based understanding of benefits calls for a large-scale distribution of the benefits of research as an important requirement of justice. This principle is relevant for political and socioeconomic critiques of the ethics of carrying on research in poor countries, given well-established patterns of exploitation and oppression of the underprivileged. Reliance on the sociological model brings out the fact that the health conditions under study originate in socioeconomic conditions that need to be treated to have an impact on the health status of research participants[28]. Thus, the notion of population or community-based benefits is related to that of health as a public good which is, in turn, linked to the global-capitalistic system that significantly contributes to the health conditions found in poor countries.

The second principle, the principle of social justice, is rooted in a broad approach to justice that places poor health at the center of public and research policy and seeks to correct systemic injustices. This principle is related to the principle of public benefit since it states that the distribution of benefits should take into account the poverty of local healthcare systems and people's disempowerment as a function of social structures[23]. Here, the challenge is that the distribution of benefits should address the root-causes of poor health and not only its symptoms. The third principle underscores the need for building local capacity. This principle states that building capacity to promote healthcare sustainability will have a lasting effect on people's health. This principle emphasizes the need for building local capacity and improving human capital to reduce the burden of preventable diseases. For example, research on AIDS vaccine often uses existing facilities or new ones built by funding agencies to conduct research or administrate the vaccine on trial. Building capacity may involve researchers and funding agencies improving the training of local medical professionals and reinforcing existing facilities to reduce the burden of disease; and, if a new medical facility has been built for the research study, local communities can still use it even after the research project comes to an end.

To avoid exploiting the underprivileged and reinforcing an existing system of oppression, the distribution of benefits should be determined by the context within which diseases occur, the state of the healthcare system, and available resources. Therefore, research institutions and their financial sponsors are morally obligated to contribute to the development of a healthcare system and the improvement of human resources that can benefit the whole population. Carrying on research in impoverished parts of the world where people have been enduring a systemic marginalization would not be ethical if our understanding of benefit will not address the root causes of poor health. Thus, it is no longer enough to avoid not doing harm; addressing health challenges that prevail in the research site is consistent with a broader view of justice[28].

### Sociological model, bioethics, and health policy

An autonomy-centered ethics places the burden of prevention and access to healthcare on the moral agent. In doing so, it frames disease within a model that limits political intervention in the health domain strictly to biomedical solutions or behavior change. This leads to the perpetuation of the social *status quo *within which risks for poor health are greater, and lends legitimacy to the social forces that increase health risks. This failure to promote social justice contrasts with John Lynch's understanding of public health intervention. Lynch believes that elements of the social fabric should shape the conception, framework, and implementation of public health intervention. Discussing the influence of socioeconomic status on behavioral and psychosocial risk factors for cardiovascular disease, he argues that the public health community should consider the potential for a broad array of social, educational, and economic policies as effective public health interventions to reduce the unequal distribution of risk factors and the unequal burden of disease[30]. Similarly, bioethicists need to study health-promoting effects of structural interventions to determine which ones are ethically acceptable and justified. Such a move requires bioethicists to look at broad issues of social equity and advocate for a shift in public policymaking.

In a population-based study examining the associations between socioeconomic status measures (education, income, and occupation) reflecting different stages of the lifecourse of 2674 middle-aged Finnish men, health behaviors, and psychosocial characteristics in adulthood, Lynch et al. conclude that: understanding that adult health behavior and psychosocial health orientations are associated with socioeconomic conditions throughout the lifecourse implies that efforts to reduce socioeconomic inequalities in health must recognize that economic policy is public health policy[31]. The sociological model within which Lynch's understanding of public health intervention is built challenges us to advocate for a shift in policymaking mindset because health is not a sphere of justice which is separate from other aspects of human life. Since disease is a social process, a policy vision that focuses on the individual and individual risk factors fails to promote social justice and to address structural elements that create conditions favorable to the production of disease. Hence, we need to move from healthcare policy to health policy, or rather, a healthcare policy that is responsive to facts explaining why (certain) people with (certain) diseases from (certain) communities require medical care. Health policy should embrace healthcare policies but include considerations regarding welfare, work, occupational, economic development, employment, and educational policies.

## Conclusion

Sociologists and social epidemiologists challenge bioethicists, especially those working in developing countries, to be socially and culturally relevant. The sociological theory of disease explanation starts with a concrete analysis of the social setting within which illness occurs or research is carried on. Since societal factors shape patterns of mortality and morbidity, principles of biomedical and research ethics need to be framed within the context of the social inequalities that shape vulnerability to illness. Aligning bioethics to perspectives, concerns and information in the fields of public health, health policy and medical sociology could vastly improve its global significance. Thus, bioethicists should be challenged to develop a philosophical anthropology that goes beyond radical affirmations of the individuality to acknowledge both the communal and the individual dimension of the human person.

## Competing interests

The authors declare that they have no competing interests.

## Authors' contributions

JA originated the article, did the research and wrote a first rough draft of the manuscript. SR read the first version and contributed editorial and critical suggestions. After the first peer-review, JA made substantial revisions to the earlier draft in close collaboration with SR. They have both read and approved the final version of the manuscript.

## Authors' informations

Jacquineau Azétsop obtained his PhD in theological and social ethics at Boston College, Massachusetts/USA and a Masters in Public Health (MPH) at the Bloomberg School of Public Health from Johns Hopkins University in Baltimore, USA. Currently, he is lecturer in health policy and bioethics at Faculté de Médécine Teilhard de Chardin in N'djaména, Chad.

Stuart Rennie (PhD, Philosophy) is currently Lecturer in the Department of Philosophy at the University of Cape Town and Research Assistant Professor in the Department of Social Medicine at the University of North Carolina (USA). He is co-Principal Investigator of UNC's Fogarty International Center bioethics capacity building project in the Democratic Republic of Congo, and ethics consultant for CDC/Global AIDS Projects in the DR Congo and Madagascar. He is co-chair of the UNC's Behavioral Institutional Review Board (IRB) and is also currently lead author of research ethics guidelines for the HIV Prevention Trials Network (HPTN). Dr. Rennie has published on research ethics and bioethics topics in PLoS Medicine, Science, the Hastings Center Report, Developing World Bioethics and the Journal of Medical Ethics, as well as writing for his own Global Bioethics Blog.
